# Configurable Encryption and Decryption Architectures for CKKS-Based Homomorphic Encryption

**DOI:** 10.3390/s23177389

**Published:** 2023-08-24

**Authors:** Jaehyeok Lee, Phap Ngoc Duong, Hanho Lee

**Affiliations:** 1 Department of Electrical and Computer Engineering, Inha University, Incheon 22212, Republic of Korea; 22211262@inha.edu (J.L.); dnphap@inha.ac.kr (P.N.D.); 2 Faculty of Computer Engineering and Electronics, The University of Danang–Vietnam-Korea University of Information and Communication Technology, Danang 50000, Vietnam

**Keywords:** homomorphic encryption (HE), Cheon-Kim-Kim-Song (CKKS), ring learning with errors (RLWE), number theoretic transform (NTT), hardware architecture

## Abstract

With the increasing number of edge devices connecting to the cloud for storage and analysis, concerns about security and data privacy have become more prominent. Homomorphic encryption (HE) provides a promising solution by not only preserving data privacy but also enabling meaningful computations on encrypted data; while considerable efforts have been devoted to accelerating expensive homomorphic evaluation in the cloud, little attention has been paid to optimizing encryption and decryption (ENC-DEC) operations on the edge. In this paper, we propose efficient hardware architectures for CKKS-based ENC-DEC accelerators to facilitate computations on the client side. The proposed architectures are configurable to support a wide range of polynomial sizes with multiplicative depths (up to 30 levels) at a 128-bit security guarantee. We evaluate the hardware designs on the Xilinx XCU250 FPGA platform and achieve an average encryption time 23.7× faster than that of the well-known SEAL HE library. By reducing time complexity and improving the hardware utilization of cryptographic algorithms, our configurable CKKS-supported ENC-DEC hardware designs have the potential to greatly accelerate cryptographic processes on the client side in the post-quantum era.

## 1. Introduction

Cloud-based computing services offer significant advantages in terms of scalability, accessibility, and cost-effectiveness. Nevertheless, they also introduce security and privacy concerns as the breach of user data can pose a significant threat. In this context, homomorphic encryption (HE) has emerged as an ideal solution to mitigate the risk of data leakage. By enabling the analysis of fully encrypted data, HE ensures the protection of users’ data from potential attacks. In an HE system, the client’s data are encrypted before being transmitted to the cloud server for computation. The cloud server performs computations on the encrypted data without any knowledge of the original data. Only the client, who holds the secret key, can decrypt the computation’s result. As a result, the employment of HE schemes ensures that the client’s data remain fully protected during analysis and transfer through the network.

The concept of computation on encrypted data was first introduced by Rivest, Adleman, and Dertouzos in 1979 [1]. Since then, numerous HE algorithms have been proposed in the literature to improve the HE system’s performance, leading to the development of practical HE schemes based on ring learning with errors (RLWE) problems [2]. However, early schemes, such as "leveled" HE, have the limitation of evaluating circuits with low depth. To overcome this limitation, Gentry introduced a bootstrapping technique that elevates leveled HE to fully HE (FHE), supporting the execution of arbitrary circuit depths by refreshing the ciphertexts. However, this technique was found to be still inefficient and expensive [3].

**Figure 1:**
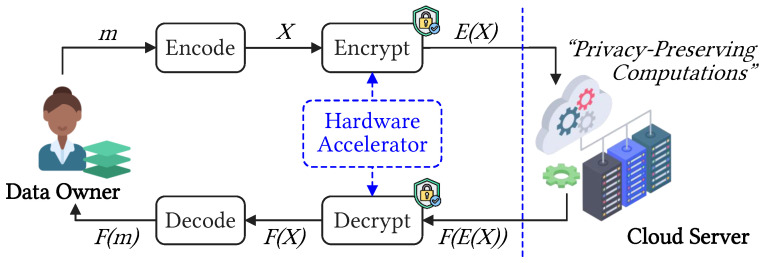
An overall view of the HE scheme with accelerated ENC-DEC operations on the client side and secure computation on the cloud server.

In the latest advancements in FHE protocols, Cheon et al. introduced the CKKS HE scheme, which enables approximate arithmetic on real or complex numbers [4]. The CKKS HE scheme has demonstrated its effectiveness in enabling the truncation of encrypted values, batch computation, and user-defined settings. As a result, many studies have investigated the application of the CKKS HE scheme for practical use cases such as privacy-preserving machine learning. As depicted in Figure 1, our main goal in this study is to design hardware architectures to accelerate CKKS-based encryption and decryption (ENC-DEC) operations on the client side. These operations are non-trivial and impose high computing and memory requirements. As the volume of data continues to grow, there is an urgent need to speed up edge-side cryptographic processes to meet the rising demands.

### 1.1. Related Works

The ENC-DEC processes are the primary bottleneck for cryptographic operations on the edge side. In the literature, there have been several studies focused on accelerating these expensive processes. Mert et al. proposed a hardware accelerator architecture for the ENC-DEC operations of the Brakerski/Fan-Vercauteren (B/FV) HE scheme in 2019 [5]. Natarajan et al. introduced SEAL-Embedded, the first HE library specifically designed for embedded devices, featuring the CKKS-based approximate HE scheme in 2021 [6]. Hagen et al. presented CHOCO, a client-optimized system for encrypted offload processing that supports both B/FV and CKKS HE schemes, in 2022 [7]. Recently, Azad et al. proposed RACE, a custom-designed, area- and energy-efficient RISC-V SoC for the data ENC-DEC using CKKS HE, in 2022 [8]. RACE unified the ENC-DEC data path and effectively utilized memory reuse and data reordering to conserve on-chip resources. Recently, Nguyen et al. proposed a high-throughput hardware architecture for CKKS-based encryption operation [9]. Their accelerator module tailored a specific parameter set of three multiplication levels and was evaluated on the Xilinx XCU250 FPGA platform. However, existing CKKS-based ENC-DEC architectures lack configuration preventing users from selecting parameters for multiple computation depths. Therefore, there is a need for a CKKS-based architecture with a flexible structure that supports various polynomial lengths and moduli, reducing the complexity of the hardware architecture and offering choices regarding precision, circuit depth, and input message size.

### 1.2. Our Contributions

To address these challenges, this study presents efficient hardware architectures for ENC-DEC accelerators that can flexibly support a wide range of polynomial sizes. Firstly, we propose configurable pipelined number theoretic transform (NTT) and inverse NTT (INTT) architectures for HE cryptosystems. These designs are capable of supporting multiple polynomial degrees, ranging from $2^{12}$ to $2^{16}$, which are commonly used in advanced HE schemes. The modular multiplier can efficiently handle arbitrary integer primes (up to a 64-bit word size) and are able to operate at high speed by optimizing the use of digital signal processing (DSP) slices. Secondly, we develop efficient hardware accelerators for ENC-DEC that can accommodate different parameter sets for the CKKS-based HE schemes. The encryption module incorporates a ModSwitch unit specifically designed to sequentially switch polynomials with various coefficient moduli. The execution of the ModSwitch unit is synchronized with other units in the encryption operation through proper scheduling. Thirdly, our experimental results demonstrate a significant average encryption speed increase of 23.7× across all tested parameters using the designed accelerator. These findings highlight the potential of our configurable CKKS-based ENC-DEC architectures to greatly enhance the performance of HE schemes and provide greater flexibility in selecting encryption parameters. Overall, this research contributes efficient hardware accelerators for CKKS-supported ENC-DEC, optimizing modular multiplication (MM) and improving the performance of HE schemes. These advancements address the challenges faced in HE implementations and pave the way for more efficient and flexible cryptographic systems on the edge.

The remainder of this paper is organized as follows: Section 2 provides the background of the CKKS-based HE operations. Section 3 presents the parameter selection in this study. In Section 4, the design method of the proposed configurable CKKS-supported ENC-DEC architectures is elaborated. Section 5 presents the evaluation results and analyzes the performance of the proposed architectures. Finally, the conclusions of this paper are summarized in Section 6.

## 2. Background

Cheon et al. introduced the CKKS HE scheme in 2017, which enables arithmetic computations on encrypted real and complex numbers [10]. The CKKS scheme operates over a quotient ring, $R_{Q}=Z_{Q}/\left(X^{N}+1\right)$, where *Q* represents a modulus integer and *N* is a power-of-two polynomial degree. The scheme encompasses four main homomorphic operations: key generation, encryption, evaluation, and decryption; while the evaluation step is typically carried out by the cloud server, most of the remaining operations are performed on the client side. Particularly, key generation involves the creation of a secret key for ENC-DEC processes conducted by a trusted party (e.g., the client user). Additionally, one or more public keys can be generated for encryption purposes or other public functional keys that will be employed during evaluation. All of these keys are derived from the underlying secret key. Encryption in the CKKS scheme is non-deterministic and can be either symmetric or asymmetric, depending on the specific requirements. Evaluation refers to performing computations on encrypted data, often carried out by an untrusted party, resulting in encrypted outputs. Finally, decryption is performed by a trusted party who possesses the secret key, allowing for the retrieval of the original plaintext data.

### 2.1. Residue Number System (RNS)

Traditionally, HE schemes operating over the quotient ring, $R_{Q}$, require the use of a large modulus integer, *Q*, to support intensive homomorphic computations. However, an efficient approach known as the Chinese Remainder Theorem (CRT) has been proposed to address this issue [11]. The CRT allows for the decomposition of the large modulus, *Q*, into smaller pairwise co-prime moduli, denoted as $q_{i}$, such that $Q=\prod_{i=0}^{L}q_{i}$. This decomposition enables the representation of a polynomial, a, in the RNS domain and facilitates efficient computations on its components. By utilizing the RNS representation, for example, the polynomial, a, can be expressed as a set of three polynomials, denoted as $a_{0},a_{1},a_{2}$, in the case of employing three pairwise co-prime moduli, i.e., $q_{0},q_{1},q_{2}$, respectively. Here, each $a_{i}$ represents a polynomial in the respective RNS channel, $R_{q_{i}}$. This technique proves advantageous as it reduces the magnitude of coefficients and significantly enhances the efficiency of arithmetic operations within the HE scheme. We denote the following polynomial components:

(1) $$a=\left({\left[a\right]}_{q_{0}},\ldots ,{\left[a\right]}_{q_{i}}\right)\in \prod_{i=0}^{L}R_{q_{i}}$$

in a ring field $R_{q_{i}}=Z_{q_{i}}$/$\left(X^{N}+1\right)$ as follows:

(2) $${\left[a\right]}_{q_{i}}=a_{0}+a_{1}X+\ldots +a_{N-1}X^{N-1}\in R_{q_{i}}$$

Therefore, performing arithmetic operations on a large integer coefficient can be executed individually for each smaller modulus without compromising precision.

### 2.2. Key Generation

The security of HE schemes, including B/FV [12,13], BGV [14], and CKKS [10], relies on the RLWE problem. In these schemes, a secret key, sk, is generated by the client using a key, s, sampled from a distribution, $\chi_{key}$, over *R*. Subsequently, a uniformly random polynomial, a, and an error polynomial, e, are generated from $U(R_{QP})$ and $\chi_{err}$, respectively. The public key, pk, is then generated as $\left(b,a\right)\in R_{QP}^{2}$, where b is obtained by taking the inner product of a and s and adding e, i.e., b=−a·s+e. Additionally, in order to switch keys in homomorphic operations (such as multiplication, permutation, and conjugation), evaluation keys are firstly generated by the client and then sent to the cloud server for further operations [4,15].

### 2.3. Enryption and Decryption Algorithms

CKKS HE scheme is based on the RLWE problem and uses approximate arithmetic instead of exact arithmetic. The scheme encodes a vector of maximal N/2 real numbers into a plaintext polynomial m of *N* coefficients with modulo *Q*. Using the generated public key, pk, the client then encrypts the input polynomials and produces a noisy ciphertext, $\mathrm{ct}=\left({\mathrm{ct}}^{0},{\mathrm{ct}}^{1}\right)\in R_{QP}^{2}$, as shown in Algorithm 1. After homomorphic computations on ciphertext on the remote side, the results are sent back to the client in the encrypted form, $\mathrm{ct}=({\mathrm{ct}}^{0},{\mathrm{ct}}^{1})$. The client uses their own secret key to decrypt the results and recover the desired information. Algorithm 2 presents the decryption process, which is performed to obtain $m^{\prime }=m+e^{\prime }\approx m$ with a small error.
**Algorithm 1** CKKS-supported encryption implementation in SEAL [16]**Input:** public key $\mathrm{pk}=\left(b,a\right)\in R_{QP}^{2}$,   encoded message $m\in R_{Q}$, vector $v\leftarrow \chi_{enc}$, errors $e_{0},e_{1}\leftarrow \chi_{err}$. **Output:** ciphertext $c=\left({\mathrm{ct}}^{0},{\mathrm{ct}}^{1}\right)\in R_{Q}^{2}$.  1:  $\tilde{v}\leftarrow$ NTT${}_{QP}\left(v\right);{\tilde{e}}_{0}\leftarrow$ NTT${}_{QP}\left(e_{0}\right);{\tilde{e}}_{1}\leftarrow$ NTT${}_{QP}\left(e_{1}\right)$  2:  ${\tilde{u}}_{QP}^{0}\leftarrow \tilde{v}\cdot b+{\tilde{e}}_{0};{\tilde{u}}_{QP}^{1}\leftarrow \tilde{v}\cdot a+{\tilde{e}}_{1}$  3:  ${\bar{u}}_{Q}^{0}\leftarrow$ ModSwitch$\left({\tilde{u}}_{QP}^{0}\right)$; ${\bar{u}}_{Q}^{1}\leftarrow$ ModSwitch$\left({\tilde{u}}_{QP}^{1}\right)$  4:  **return** $c=\left({\mathrm{ct}}^{0},{\mathrm{ct}}^{1}\right)=\left({\bar{u}}_{Q}^{0}+m,{\bar{u}}_{Q}^{1}\right)$
**Algorithm 2** CKKS-supported decryption implementation in SEAL [16]**Input:** ciphertext $c=\left({\mathrm{ct}}^{0},{\mathrm{ct}}^{1}\right)\in R_{Q_{l}}^{2}$,   secret key sk=(1,s). **Output:** approximate message $m^{\prime }$  1:  $m^{\prime }\leftarrow {\mathrm{ct}}^{1}\cdot s+{\mathrm{ct}}^{0}$  2:  **return** $m^{\prime }\left(\approx m\right)$

## 3. Recommended Parameters for HE Schemes and Our Selection

In contrast to post-quantum cryptography (PQC), RLWE-based HE techniques are supported by multiple standards offering various parameter sets for different circuit depths with a given security level [17]. The HE standard provides a table of recommended parameter sets that users can select based on their desired security level, precision requirements, and circuit depth of computations. The parameter λ determines the security level against attacks, with higher values providing stronger security but reducing the total bit size of the modulus. The performance of the HE system is determined by crucial parameters including the polynomial degree and ciphertext modulus. Accordingly, increasing the polynomial degree, *N*, results in larger ciphertext sizes, enabling the utilization of larger modulus bit sizes and supporting more complex homomorphic operations. However, this comes at the cost of slower operation speed and increased hardware consumption. Therefore, the selection of appropriate parameter sets is crucial for each specific use case. Table 1 presents the recommended parameter sets from [17] with an error standard deviation of σ≈3.2. The security level, λ, can be guaranteed if the user performs operations with the specified security parameter, which is mainly determined by setting the polynomial degree and modulus bit size for a desired circuit depth.

In this study, we carefully selected the parameter sets for the proposed configurable architectures of CKKS-based ENC-DEC modules. The selection of these parameters was guided by the desired security levels, the bit precision when decrypting, and the multiplicative depth required for performing computations on ciphertexts. We meticulously determined the parameter setting to ensure a comprehensive assessment of all parameter variations. Our approach aligns with the standard set by the HE standardization group [17] and the parameter configuration utilized in Microsoft SEAL HE library [16]. We opted for parameter sets that encompass the maximum supported level corresponding to the polynomial degree, *N*. Accordingly, we guarantee a security level of λ = 128 bits by appropriately setting the bit sum of the moduli and their associated *N* value. The right section of Table 1 points out the chosen parameter sets that were utilized to implement the configurable architectures in this study.

## 4. Proposed Configurable CKKS-Supported ENC-DEC Architectures

This paper primarily focuses on the design of configurable accelerators for ENC-DEC operations. These hardware architectures are specifically designed to support a broad spectrum of polynomial degrees (ranging from $2^{12}$ to $2^{16}$) associated with up to 32 moduli. This configurability enables the accelerators to adapt to different computational depths through compile-time configuration. To provide a thorough understanding of the hardware designs, we first introduce the design methodology of the configurable NTT and INTT modules, which play a vital role as key computational components within the ENC-DEC accelerators. The subsequent sections delve into the architectural details of these designs, shedding light on their functionality and implementation.

### 4.1. Proposed Configurable NTT and INTT Architectures

**Figure 2:**
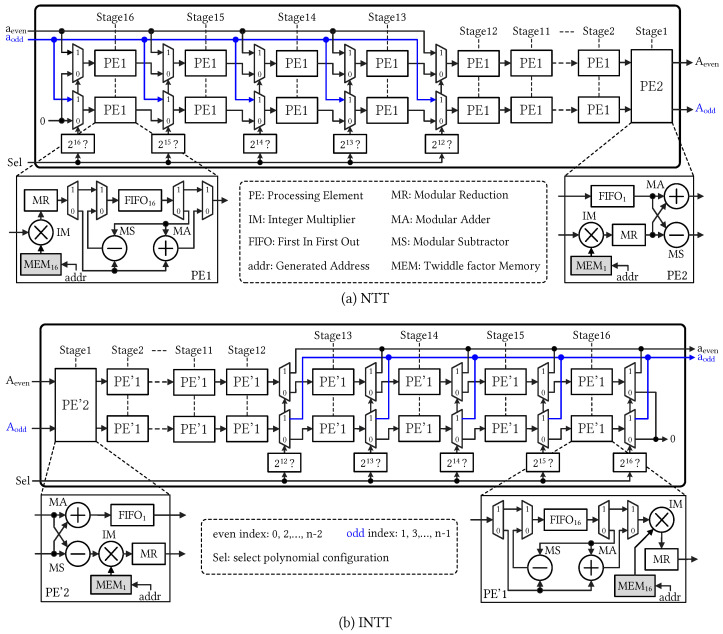
Proposed configurable two-parallel MDF architectures for (**a**) Cooley–Tukey decimal-in-time NTT and (**b**) Gentleman–Sandy decimal-in-frequency INTT accelerators supporting various polynomial degrees, *N*, from $2^{12}$ to $2^{16}$.

Figure 2 illustrates the architecture design of the fundamental computational units, namely NTT and INTT, essential for accelerating expensive polynomial multiplication in HE schemes. These units are constructed using two-parallel multi-path delay feedback (MDF) architecture, which enables NTT and INTT executions to generate coefficients every clock cycle in a fully pipelined manner. Different from a prior study [18], we targeted various polynomial degrees and larger integer moduli for practical HE schemes. The proposed NTT and INTT units are configurable to support various polynomial degrees by manipulating the multiplexer selection signal. The modular computation units were designed to support arbitrary moduli with a wide range of bit width, i.e., from 20 to 64 bits.

The two-parallel MDF NTT computational unit can support multiple polynomial degrees from $2^{12}$ to $2^{16}$ through compile-time configuration. As shown in Figure 2a, the input data are divided into two paths of $a_{even}$ and $a_{odd}$, corresponding to the even and odd coefficient indices, respectively. After a propagation delay, each pair of two coefficients are produced every clock cycle. The NTT unit transforms consecutive polynomials in a fully pipelined manner. To support the largest polynomial degree of $N=2^{16}$, the NTT unit performs 16 computational stages, and the signal, *Sel*, selects the desired configuration through multiplexers. Processing elements (PEs) perform underlying computations in butterfly operations across stages. With the two-parallel data path, the first 15 stages employ PE1 and the last stage uses PE2. Detailed architectures of PE1 and PE2 are zoomed out in the figure. A feedback operation was performed in PE1 using the first-in-first-out (FIFO) registers. Twiddle factors (TFs) were pre-calculated and stored in the internal memory for integer multiplication. The same design methodology was employed for the INTT architecture. In contrast to the NTT operation, the INTT exhibits a mirror-symmetric data flow, as shown in Figure 2b. Consequently, the INTT unit executes the stages in reverse order compared to the NTT operation. The first stage of the INTT unit employs PE2, and the deployment of PE1s in the entire INTT structure is adjusted accordingly.

**Figure 3:**
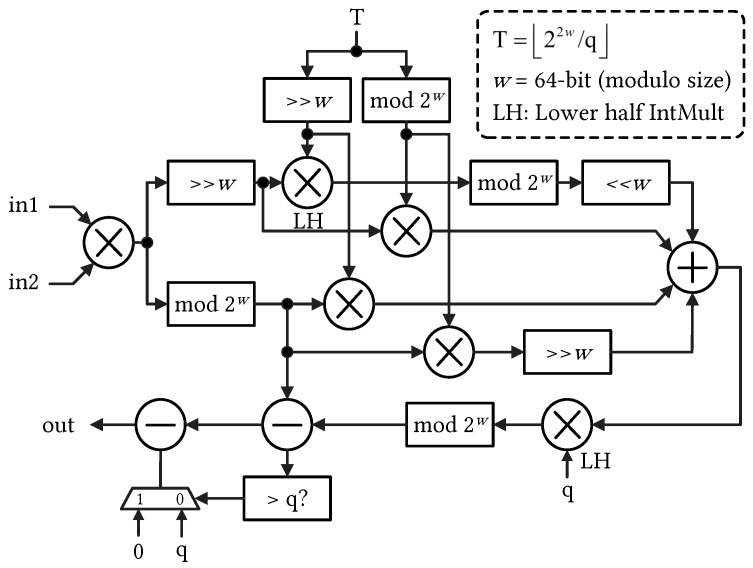
Modular multiplier supporting arbitrary 64-bit modulo primes (derived from [16]).

The MM between input polynomial coefficients and TF constants is the most computationally intensive task in the PEs. To achieve a high-performance MM operation, this study leveraged an efficient Barrett-based MM algorithm with the use of DSP slices. The architecture of the modified MM unit is illustrated in Figure 3. The MM unit is specifically designed to handle arbitrary word-size integers up to 64 bits. Within the MM unit, each of the 4 full integer multipliers utilizes 12 DSP slices, while each of the 2 half-integer multipliers requires 8 DSPs. Due to the limitation of the supported input size, 64-bit integers are decomposed into smaller parts, and the DSP slices manage partial multiplication and multiply–accumulate operations to generate the correct outputs. As a result, a total of 64 DSP slices are consumed within each MM unit.

### 4.2. Proposed Configurable ENC-DEC Architectures

**Figure 4:**
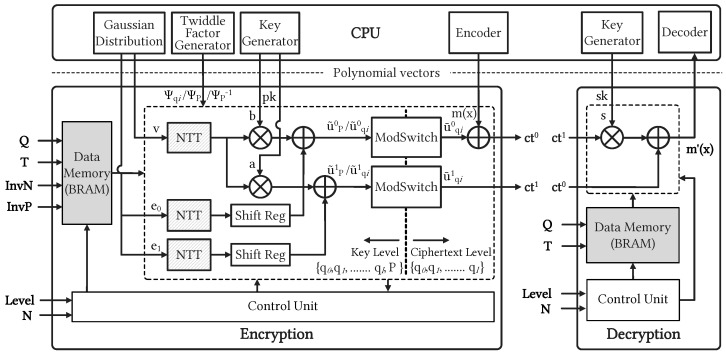
System-level view of the cryptosystem encompassing the proposed CKKS-supported configurable ENC-DEC accelerators.

Figure 4 illustrates the CKKS-based HE cryptosystem employing the proposed ENC-DEC accelerators. The parameters of various configurations (such as modulus, *Q*, and the pre-calculated values of *T*, InvN, and InvP) were first loaded into the internal memory for compile time. The desired computational level, Level, was configured with the selection of *N* and *Q* through *Control Unit* (CU). Level denotes the number of available homomorphic operations and helps manage the noise that accumulates during these operations. For instance, if Level is 2, four modulus integers are utilized, as shown in Table 1. The ratio value, *T*, was pre-calculated and used for the Barrett-based MM algorithm. InvN and InvP represent the inverse modulo of *N* and special prime, *P*, respectively. The *Data Memory* (DM) unit stores essential constants and allows access to data through the reading signal generated by the CU. The CU receives the parameters of Level and *N* and configures the encryption module accordingly.

The ENC-DEC modules work in conjunction with the CPU to facilitate the homomorphic operations on the client side. Initially, the CPU generates and stores various values, such as a random vector (v), noise vectors ($e_{0}$, $e_{1}$), public key (pk=(b,a)), TF constants ($\Psi_{q_{i}}$, $\Psi_{P}$, $\Psi_{P}^{-1}$), and plaintext (m(x)), in memory to be utilized by the proposed accelerator modules. As illustrated in Figure 4, the stored values, v, undergo consecutive NTT${}_{P}$ and NTT${}_{q_{i}}$ operations, followed by multiplication with the public key, pk. Subsequently, the results of the multiplication are added to the corresponding outputs of NTT${}_{P}$ and NTT${}_{q_{i}}$ of $e_{0}$ and $e_{1}$, respectively. The resulting vectors, ${\tilde{u}}^{0}$ and ${\tilde{u}}^{1}$, are then passed through the ModSwitch unit to rescale them with the special prime, *P*. Finally, the ciphertext values ($\mathrm{ct}=({\mathrm{ct}}^{0}$, ${\mathrm{ct}}^{1})$) are obtained by adding the encoded plaintext, m(x), to the output of the ModSwitch unit. The TF constants ($\Psi_{q_{i}}$, $\Psi_{P}$, $\Psi_{P}^{-1}$) are generated by the CPU and appropriately fed into the NTT and INTT units as required.

#### 4.2.1. ModSwitch Unit

**Figure 5:**
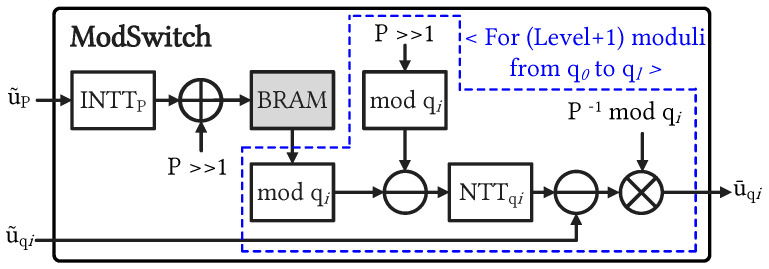
Detail block diagram of the ModSwitch module, which serially switches the polynomial of a special modulus (P) to the data level (i.e., ciphertext polynomials of scaling modulus primes).

In RLWE-based HE protocols, ModSwitch module is essential for switching data from the key level to the ciphertext level. Figure 5 depicts the block diagram of a versatile ModSwitch architecture supporting configurable *N* and Level. In the ModSwitch operation of (Level + 2), the INTT unit operates only once for the polynomial of modulus *P*, a modular addition operation with (P>>1), and we then use a DM unit to store the INTT result for the following operations. Particularly, the blue-dot box in Figure 5 indicates sequential (Level + 1) operations on moduli $q_{i}$ associated with the previous ${\tilde{u}}_{q_{i}}$ results. With the configuration of the parameter set (*N* and Level), the CU schedule data read and write the operations of the DM unit. During the ModSwitch operation, the CU selectively retrieves the required parameter from the DM unit for the corresponding configuration. The CU manages the write signal of the BRAM block and schedules its reading operation for the following computations on $q_{i}$. In CKKS-based HE systems, the selected modulus plays a critical role in determining the size of residue channels and influencing the precision of homomorphic computation results. ModSwitch facilitates efficient conversion between moduli, ensuring the confidentiality of the underlying plaintext while achieving the desired precision or reducing noise in computations.

#### 4.2.2. Controller and Timing Diagram

**Figure 6:**
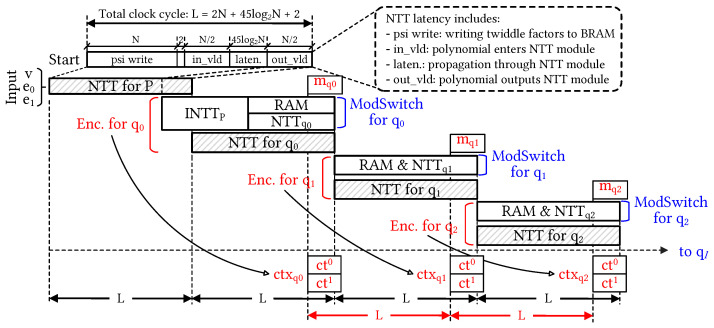
Timing diagram of the encryption acceleration. The point-wise multiplication with public keys and the following addition are eliminated for the sake of simplicity.

The CU plays a crucial role in orchestrating the synchronization and scheduling of all sub-units within the ENC-DEC modules. In the encryption module, the configurable architecture offers flexibility for various values of *N* and Level by utilizing a MUX to determine the *N* value and repeating the encryption operation (Level + 2) times. It is important to notice that the first step of the ModSwitch module reduces the bit width of special primes. This occurs because the ModSwitch module converts ciphertexts from the key level to the data level by extracting it from the special prime. The polynomial channel corresponding to the special prime enters the INTT${}_{P}$ unit and is then temporarily stored in the BRAM for subsequent operations, as indicated by the blue-dot box in Figure 5. The configurable execution of the encryption module is primarily governed by the pipelined NTT and INTT units with various selectable configuration modes. In contrast to the previous approach [9], which employed multiple NTT units in parallel, our configurable encryption design is scheduled to store temporary data in the BRAM and perform multiple NTT operations with associated moduli in a pipelined manner. To facilitate this, we established a counter with signal flags for each stage of the NTT and INTT operations. The CU determines which polynomial degree is configured by selecting the appropriate MUXs. Additionally, the control mechanism in the decryption module is simpler, primarily involving integer multiplication and addition operations. Figure 6 illustrates the timing diagram of the configurable CKKS-based encryption operation, providing a comprehensive view of the computational process in the time domain. Notably, the grey striped blocks represent three NTT modules that simultaneously transform input vectors v, $e_{0}$, and $e_{1}$ in parallel. The latency of the encryption process is determined by the total computation time of (Level + 2) NTT operations. As the Level increases, the encryption operation is more time-consuming. The change in computational Level is executed through a series of repeated encryption operations, which occur (Level + 2) times. The number of clock cycles required for the encryption modules, including NTT, multiplier, adder, and ModSwitch, follows a specific rule as *N* increases. The CU adjusts the number of cycles for each module based on the input value of *N*. This enables the encryption of plaintext supporting a wide range of *N* from $2^{12}$ to $2^{16}$. Notably, the encryption module heavily relies on the pipelined execution of NTT units to synchronize whole operations. In the design methodology, each NTT operation with prior TF writing has a cycle latency function denoted as *L*, defined by the following equation:

(3) $$L=2N+45\log_{2}N+2$$

For example, in the case of *N* = 16,384, one encryption operation is performed at every count value of 33,400. A total of (Level + 1) ModSwitch operations are repeated, with each operation governed by the function L for the given parameter *N*.

## 5. Evaluation Results and Discussion

Verilog HDL implementation of the proposed configurable CKKS-based ENC-DEC accelerators was carried out using the Xilinx Vivado${}^{\mathrm{TM}}$ (v2020.2) tool. We then performed the logic synthesis to convert the register-transfer level design into a netlist comprising primitive FPGA logic elements. The synthesized netlists were subsequently deployed on the Xilinx Alveo UltraScale+ XCU250 FPGA platform. The utilization of on-chip resources and the maximum clock frequency were obtained using the default settings. This evaluation provided insights into the efficient utilization of FPGA resources and the achievable performance of the implemented accelerators.

Table 2 presents the comprehensive FPGA resource breakdown for the configurable NTT and INTT hardware modules. For a fully pipelined MDF implementation, these modules consume a large amount of FFs for FIFO operations in the BU structure. Additionally, the on-chip resource utilization of sub-modules is also reported. In which, MM is the main unit and consumes 64 DSP slices for arbitrary 64-bit integers. Consequently, the two-parallel MDF NTT module in Figure 2 with 16-stage configuration uses a number of 1984 (=64×15+64) DSP slices. For the INTT module, the scaling operation with $N^{-1}$ at the last stage uses an additional 128 DSP slices. Therefore, the INTT module consumed a total number of 2112 (=1984+128) DSP slices. All modules are designed to run at 250 MHz clock frequency. The last column reports the number of consumed CCs for the corresponding modules.

In Table 3, we compare the effectiveness and performance of the proposed NTT architecture design with related works. We calculate equivalent slices and use hardware efficiency metrics for a fair comparison across studies. The hardware efficiency is evaluated by measuring the throughput rate per equivalent slice. We focus on a direct comparison with studies [19,20], which primarily utilized pipeline-based design methods. In [19], Ye et al. proposed PipeNTT, a parametric single-path delay feedback (SDF) radix-2 NTT architecture, for PQC systems. PipeNTT employed both BRAM and FF to rearrange intermediate coefficients. With a similar parameter configuration, our NTT design could achieve an approximately 5.89× larger throughput and 73% greater hardware efficiency compared to that of [19]. Additionally, Hirner et al. [20] proposed a parametric tool to generate NTT architectures for user-specified parameter sets. They targeted MDC-based design for high-performance and bandwidth-efficient NTT implementation. Meanwhile, our MDF-based architecture features a simpler controller with configurability and high-performance implementation. We also achieved a 1.65× higher throughput and 21% greater hardware efficiency than that of [20], despite higher hardware resource consumption. On the other hand, Kurniawan et al. proposed the latest configurable memory-based NTT architecture with less hardware resource consumption [21]. However, our design consumes more DSP slices due to supporting arbitrary modulus primes. This study also utilizes a pipelined NTT architecture to accelerate the encryption operation through efficient scheduling. To this end, Table 3 shows that our NTT design can achieve a higher throughput rate and better efficiency than [19,20] and has comparable performance compared with the configurable iterative architecture in [21].

Table 4 provides a comprehensive performance comparison of the configurable CKKS-based ENC-DEC modules with the SEAL HE library. We measured the number of operations per second for various parameter sets. The comparison results indicate that our configurable CKKS-based ENC-DEC modules consistently outperform the SEAL library, achieving an average performance improvement of 23.7× for encryption time and 10.9× for decryption time across various parameter configurations. The significant speedups achieved underscore the pipelined implementation and efficient scheduling of our designs. The superior performance of our ENC-DEC modules enables faster and more efficient HE operations, making them highly suitable for practical applications that demand both security and performance.

Table 5 showcases the implementation results of the configurable CKKS-based ENC-DEC hardware modules, providing insights into the resource utilization and performance of both modules. These designs were successfully accelerated on the Xilinx Virtex UltraScale+ XCU250 FPGA platform, operating at a clock frequency of 250 MHz for high-speed processing. The encryption module utilizes hardware resources of 1179K LUT elements, 1036K FFs, 12,288 DSP slices, and 828.5 BRAM units. In contrast, the decryption module exhibits even more optimized resource utilization, employing 10.7K LUT elements, 6.9K FFs, 133 DSP slices, and 3 BRAM units. A similar study on accelerator architecture for CKKS-based encryption operations can be found in [9]. However, their hardware design supports only a specific parameter set of ($N=2^{14}$, Level=3), while our encryption accelerator is configurable to accommodate varying circuit depths. We can estimate that deploying multiple NTT modules in parallel as [9]’s approach for higher polynomial sizes would considerably consume hardware resources. Conversely, our method deploys a single NTT hardware module and effectively schedules NTT operations for different moduli in a pipelined manner.

Our implementation results confirm the feasibility of the configurable CKKS-based ENC-DEC hardware designs with efficient hardware resource consumption. These results emphasize the scalability and effectiveness of the proposed designs, achieving a harmonious balance between resource utilization and performance. Numerous practical applications can benefit from CKKS-based ENC-DEC accelerators on the client side. For instance, a sensor-based image/video or sensitive financial and medical data could be encrypted locally before being shared with other devices or cloud services for analysis. These applications highlight the potential of the proposed designs in facilitating machine learning tasks integrated with HE while maintaining data privacy and security.

## 6. Conclusions

We presented novel hardware architectures for configurable CKKS-based ENC-DEC accelerators. These accelerator modules have the capability to support a wide range of parameter sets, including different polynomial lengths and multiplicative depths, making them suitable for practical CKKS HE cryptosystems. The experimental results demonstrate that our hardware designs achieved high data throughput during ENC-DEC processes. Consequently, the proposed hardware designs are expected to significantly accelerate homomorphic computations on the client users. Future studies will investigate HE-integrated machine learning algorithms and leverage the presented acceleration techniques to enable the deployment of advanced HE systems tailored for privacy-preserving applications.

## Figures and Tables

**Table 1:** Recommended parameters (polynomial degree, *N*, and modulus bit length, logQ) for given security levels, λ, and the selected parameter sets for this study (with λ= 128 bits).

N	Recommended Parameter [17] (logQ)			Selected Parameter	
	λ= 128 bits	λ= 192 bits	λ= 256 bits	Level	Coefficient moduli (bits)
$2^{12}$	109	75	58	2	{30, 20, 20, 30}
$2^{13}$	218	152	118	5	{30, 20, 20, 20, 20, 20, 30}
$2^{14}$	438	305	237	6	{60, 48, 48, 48, 48, 48, 48, 60}
$2^{15}$	881	611	476	15	{60, 48 (×15)…, 60}
$2^{16}$	1782	1242	963	30	{60, 48 (×30)…, 60}

**Table 2:** FPGA resource utilization of the configurable NTT and INTT hardware modules.

Module	Resource Utilization				Freq.	Latency
	LUT	FF	DSP	BRAM	(MHz)	(CC)
NTT	59,619	109,226	1984	240.5	250	33,488
⌊ MM	1463	3339	64	0	-	42
⌊ MA	195	0	0	0	-	1
⌊ MS	97	0	0	0	-	1
INTT	65,291	115,323	2112	240.5	250	33,529

**Table 3:** NTT implementation result and performance comparison with related studies.

Design	N	Max $q_{i}$	FPGA Resource Utilization					Freq.	Latency	Thro.	Effi.	Device
		(bits)	LUT	FF	DSP	BRAM	E.S ^‡^	(MHz)	(μs)	(Mbps)	($\frac{\mathrm{Mbps}}{E.S}$)	
[19]’22	$2^{12}$	60	17K	11K	286	100	38.7K	150	55	4450	0.11	XV485
[20]’23	$2^{16}$	64	31.3K	24.4K	300	255	97.8K	150	221	19,009	0.19	XV485
[21]’23	$2^{16}$	60	74.5K	61.4K	288	155	49.7K	250	66.5	59,120	1.19	VU37P
	$2^{12}$								**10**	**26,214**	**0.19**	
	$2^{13}$								**19**	**27,594**	**0.20**	
**Ours**	$2^{14}$	**64**	**59.6K**	**109.2K**	**1984**	**240.5**	**137K**	**250**	**35**	**29,959**	**0.22**	**XCU250**
	$2^{15}$								**68**	**30,841**	**0.23**	
	$2^{16}$								**134**	**31,301**	**0.23**	

^‡^ Equivalent Slices (ES) (=#Slice + E.DSP + E.BRAM) is calculated as in [15].

**Table 4:** Performance comparison (in terms of the number of operations per second (ops)) of the configurable CKKS-supported ENC-DEC modules with the SEAL HE library [16].

Parameter		Encryption (ops)		Decryption (ops)	
N	Level	Intel Core-i7	XCU250 (Speedup)	Intel Core-i7	XCU250 (Speedup)
$2^{12}$	2	377	14,493 (×38.4)	16,631	62,500 (×3.8)
$2^{13}$	5	123	2950 (×24.0)	4460	12,195 (×2.7)
$2^{14}$	6	60	1249 (×20.8)	1686	5076 (×3.0)
$2^{15}$	15	13	251 (×19.3)	379	1015 (×2.7)
$2^{16}$	30	4	64 (×16.0)	6	254 (×42.3)

**Table 5:** Hardware utilization and performance of the proposed configurable ENC-DEC accelerators evaluated on the Xilinx Virtex UltraScale+ XCU250 FPGA platform.

Parameter	Encryption		Decryption
Design	[9]’23	This work	This work
N	$2^{14}$	$2^{12}\;∼\;2^{16}$	$2^{12}\;∼\;2^{16}$
Level	3	2∼30	2∼30
LUT	883K (51%)	1179K (68%)	10.7K (0.6%)
FF	897K (26%)	1036K (30%)	6.9K (0.2%)
DSP	6042 (58%)	12,288 (100%)	133 (1%)
BRAM	1563 (49%)	828.5 (31%)	3 (0.1%)
Freq. (MHz)	250	250	250
Latency (μs)	102.1	16,869 ^§^	3937 ^§^

^§^ Our results are measured for parameter set of $N=2^{16}$ and Level=30.

## Data Availability

Not applicable.
